# Supplementary material for: Chirality transfer from a 3D macro shape to the molecular level by controlling asymmetric secondary flows
Source: Nat Commun. 2022 Apr 1;13:1766. doi: 10.1038/s41467-022-29425-y (PMC8976054; doi:10.1038/s41467-022-29425-y)
Supplement: Supplementary file 1 — Suplementary Information [file 41467_2022_29425_MOESM1_ESM.pdf]

Supplementary Information

Chirality transfer from a 3D macro shape to the molecular level by controlling asymmetric secondary flows

Semih Sevim,†[1],[2] Alessandro Sorrenti,†*[1],[3],[4] João Pedro Vale,†[5],[6] Zoubir El-Hachemi,[3] Salvador Pané,[2] Andreas D. Flouris,[7] Tiago Sotto Mayor,*[5],[6] Josep Puigmartí-Luis*[4],[8],[9]

† Contributed equally to this work

[1]*Institute of Chemical and Bioengineering, Department of Chemistry and Applied Biosciences, ETH Zurich, Zurich 8093, Switzerland*

[2]*Multi-Scale Robotics Lab, ETH Zurich, Tannenstrasse 3, CH-8092 Zurich, Switzerland.*

[3]*Departament de Química Inorgànica i Orgànica (Secció de Química Orgànica), University of Barcelona (UB), 08028 Barcelona, Spain*

[4]*Institut de Química Teòrica i Computacional, University of Barcelona (UB), 08028 Barcelona, Spain*

[5]*Transport Phenomena Research Centre (CEFT), Engineering Faculty of Porto University, Rua Dr. Roberto Frias, 4200-465 Porto, Portugal*

[6]*Associate Laboratory in Chemical Engineering (ALICE), Engineering Faculty of Porto University, Rua Dr. Roberto Frias, 4200-465 Porto, Portugal*

[7]*FAME Laboratory, Department of Exercise Science, University of Thessaly, Greece*

[8]*Departament de Ciència dels Materials i Química Física, University of Barcelona (UB), 08028 Barcelona, Spain*

[9]*Institució Catalana de Recerca i Estudis Avançats (ICREA), Pg. Lluís Companys 23, 08010 Barcelona, Spain*

Email: *asorrenti@ub.edu, tiago.sottomayor@fe.up.pt, josep.puigmarti@ub.edu*

Geometrical parameters and design of the used 3D-printed fluidic devices

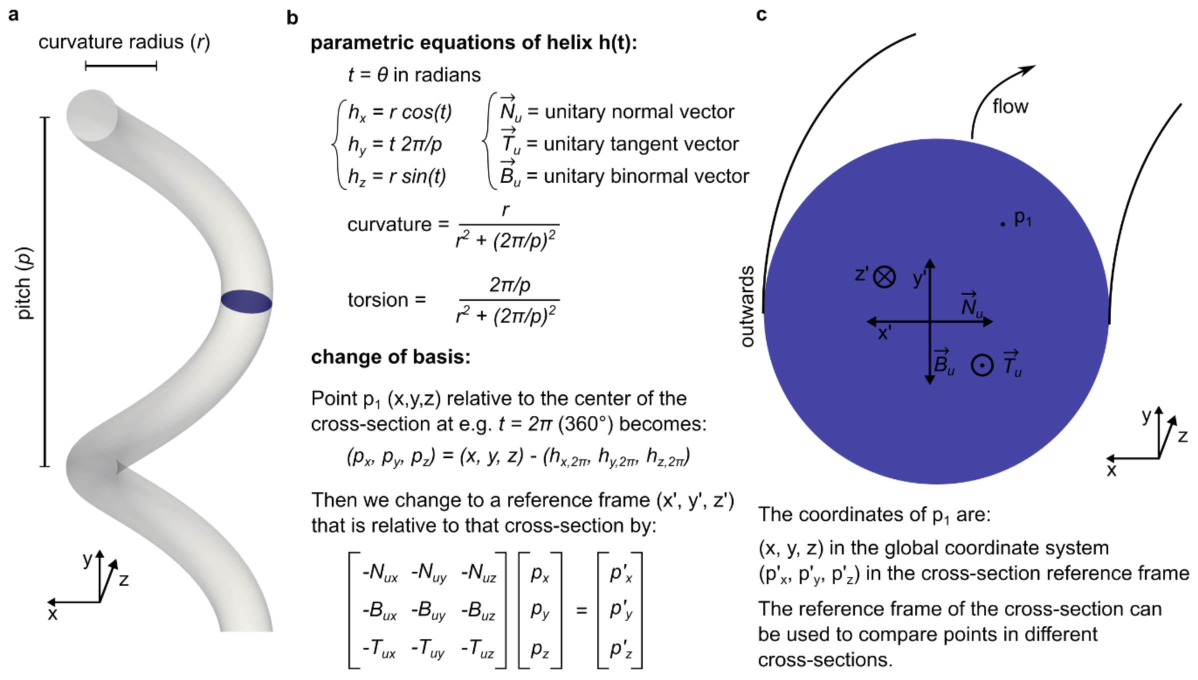

Figure 1: Change of basis to a reference frame that is relative to each cross-section. a, Representation of the curvature radius (r) and pitch (p) of an helix. **b,** Description of the parametric equations of a helix, and method of change of basis that was used to shift the coordinates of points from the global coordinate system to a coordinate system that is relative to each cross-section. **c,** Representation of the coordinate system relative to each cross-section (x', y', z') with which the results at different cross-sections can be compared.

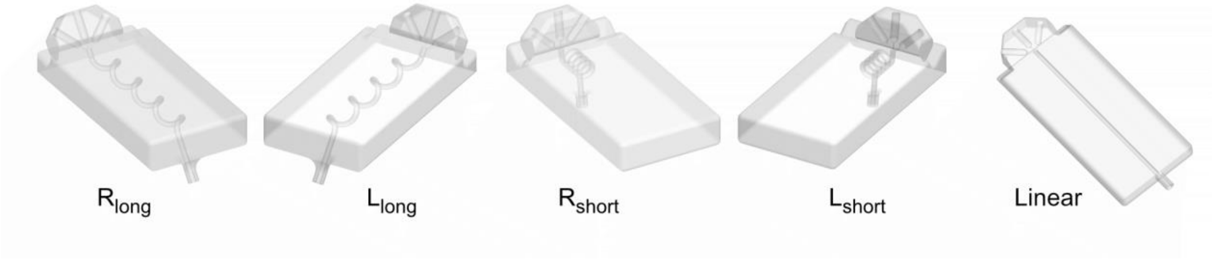

Figure 2. 3D illustration of the devices used in this work. Short-and long-pitch helical devices with 1.5 mm and 7.5 mm pitch, respectively, in both their R- and L-enantiomeric forms (comprising right-handed and left-hand ed helical channels respectively). A linear device comprising a straight channel. The channels in all the devices have a cross-section diameter of 1mm.

Table 1. Geometrical parameters for different kinds of microfluidic channels used for experiments.

	**Pitch (mm)**	**Cross-sectional diameter (mm)**	**Length of outlet part (mm)**	**Radius of curvature (mm)**	**Number of turns**
**Short-pitch**	1.5	1	6	1.5	4
**Long-pitch**	7.5	1	6	1.5	4
**Linear**	÷	1	6	***n.a.***	***n.a.***

Visualization of the secondary flow: fluid trajectories

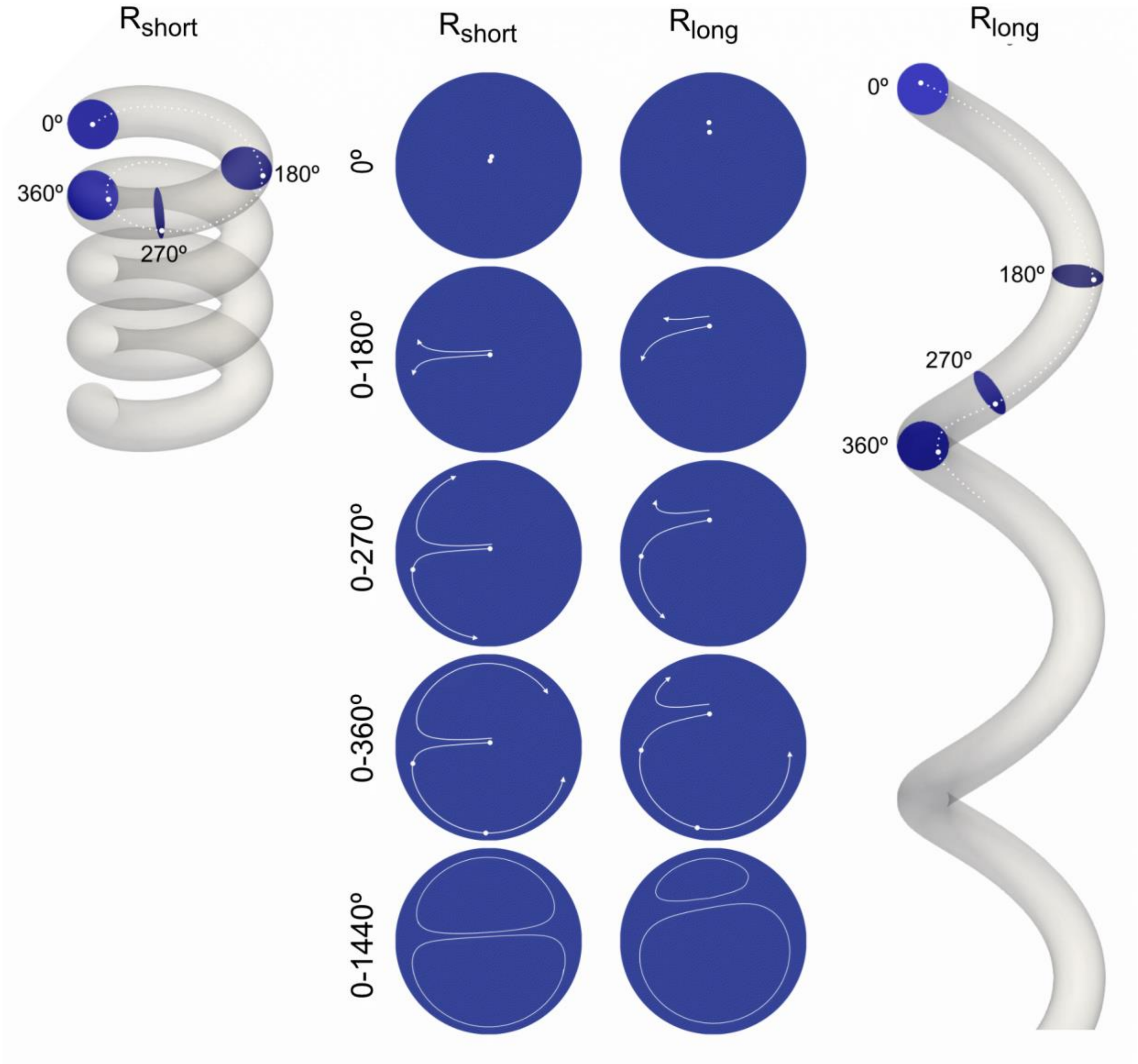

Figure 3. Construction of the fluid trajectories. The fluid trajectories are obtained by connecting the points that represent the position of selected fluid element with respect to a reference frame centered in each cross-section, as those fluid elements move downstream in the channel (i.e., as the fluid flows along the helix). All the positions measured with respect to each cross-section are then represented on a single cross-section. In this representation, the vortices are a visualization of the helical twist that the streamlines (i.e. the dashed white lines shown in the lateral helixes) experience in the helical channel.

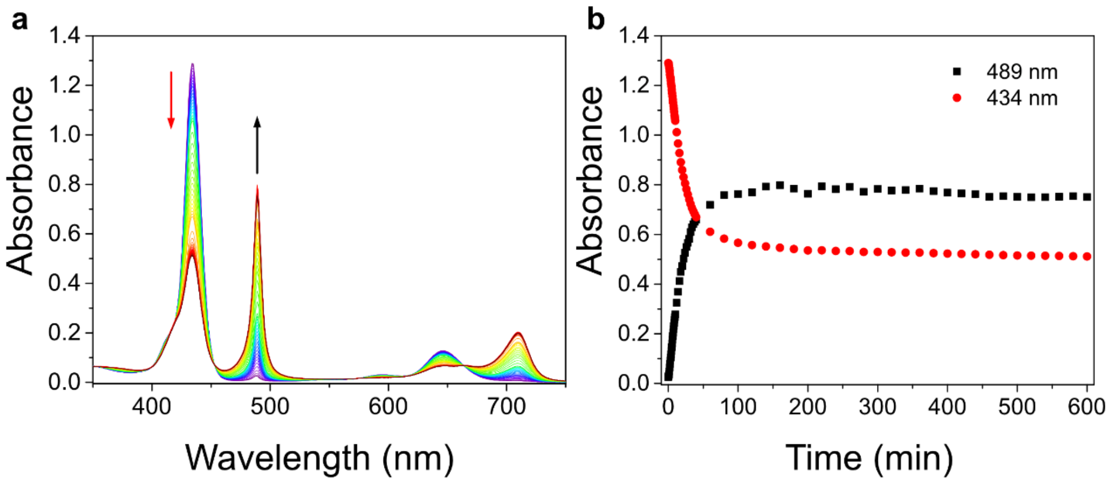

Figure 4: Time course UV-Vis spectra. a, Representative time-course UV-Vis spectra and **b,** plot of the absorbances at 434 and 489 nm over time for a solution eluted from an helical device. The figure shows the incipient formation of J-aggregates in the just eluted solution (within 5 min), and further aggregation of the protonated porphyrin occurring out-of-chip in the following 3-4 hours after elution.

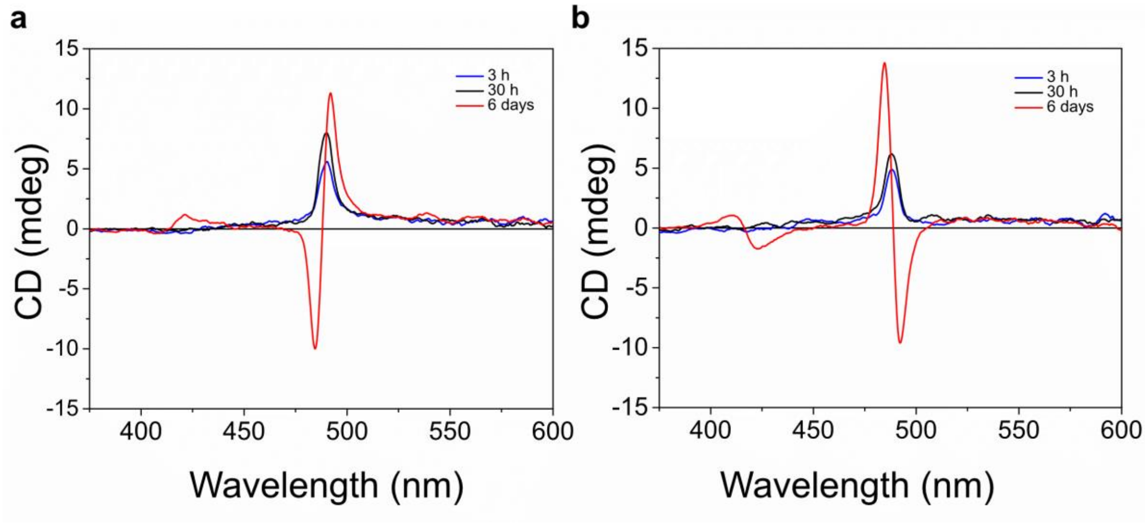

Figure 5: Time course CD spectra. Evolution of the CD spectra recorded at 3 h, 30 h and 6 days showing the development of bisignate CD bands upon ageing in representative samples: **a**, prepared using the R_{long} device and **b,** prepared using the L_{long} device.

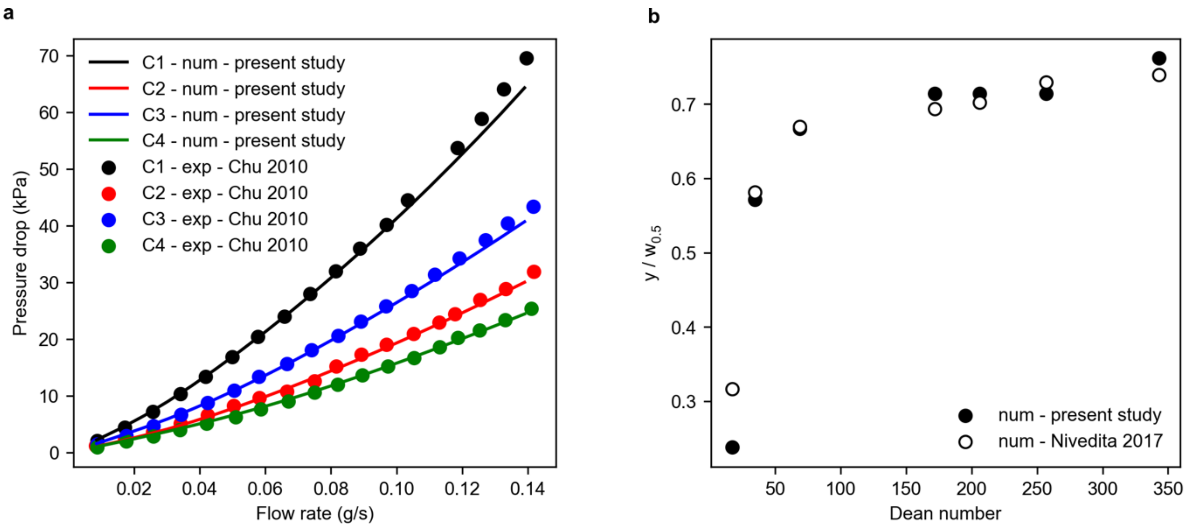

Figure 6: Predictions by the present numerical model versus experimental and numerical literature data. a, Comparison of the curves of pressure drop vs flow rate that were experimentally obtained by Chu et al. 2010 for different curved microchannels (C1-C4), with the results obtained with the present numerical approach for the same conditions. **b,** Comparison of the shift in the position of the point of maximum velocity from the centre of the channel (y) relative to half of the channel width ($w_{0.5}$), obtained with the present numerical approach for a spiral microchannel with different Dean numbers, relative to those numerically obtained by Nivedita et al. 2017.

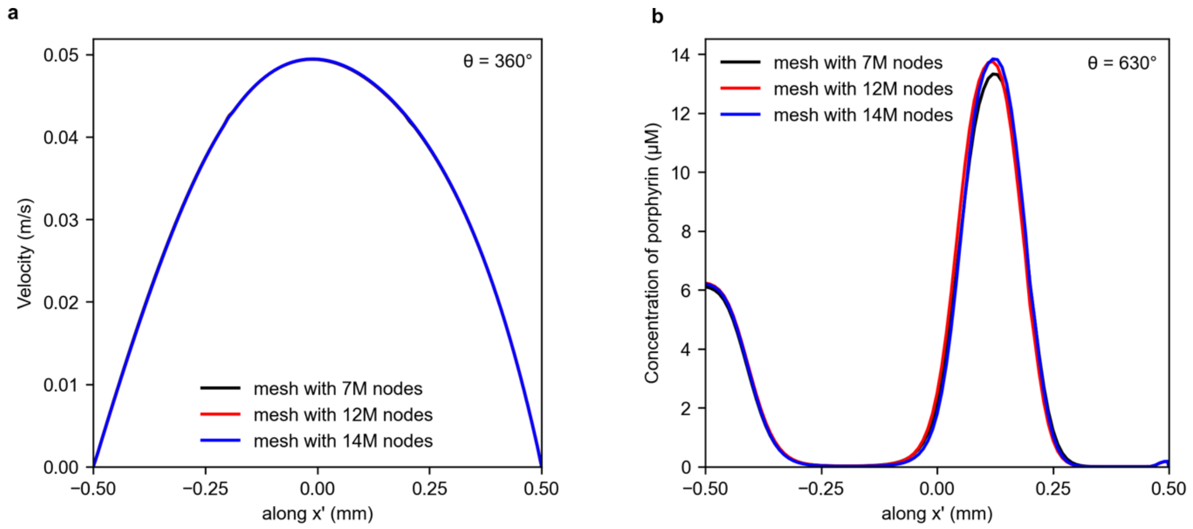

Figure 7: Mesh independence testing. a, Velocity profile at θ = 360° and **b,** concentration profile at θ = 630° along x' for meshes of increasing complexity (7, 12 , and 14 million nodes) for R_{short}. These representative profiles show that a mesh containing 12 million nodes produces results that are similar to those obtained with a denser mesh. For this reason, a mesh with 12 million nodes was used for all the numerical analyses.
