# Supplementary material for: Chirality transfer from a 3D macro shape to the molecular level by controlling asymmetric secondary flows
Source: Nat Commun. 2022 Apr 1;13:1766. doi: 10.1038/s41467-022-29425-y (PMC8976054; doi:10.1038/s41467-022-29425-y)
Supplement: Supplementary file 3 — Description of Additional Supplementary Files [file 41467_2022_29425_MOESM3_ESM.pdf]

Description of Additional Supplementary Files

File Name: Supplementary Movie 1

Description: Mass transport in short- and long-pitch devices. Concentration distribution of $TPPS_3$ along the entire length of the helical channel for R_{short} and R_{long} devices (left and right respectively). The concentration distribution evolves in a markedly different way in the two devices, with the porphyrin remaining exposed exclusively to the CCW vortex in R_{long} and to both the CCW and the CW vortices in R_{short}.
